# Hybrid Pd-Nanoparticles within Polymeric Network in Selective Hydrogenation of Alkynols: Influence of Support Porosity

**DOI:** 10.3390/molecules27123842

**Published:** 2022-06-15

**Authors:** Linda Z. Nikoshvili, Alexander Y. Popov, Alexey V. Bykov, Alexander I. Sidorov, Lioubov Kiwi-Minsker

**Affiliations:** 1 Department of Biotechnology, Chemistry and Standardization, Tver State Technical University, A. Nikitina Str., 22, 170026 Tver, Russia; bykovav@yandex.ru (A.V.B.); sidorov@science.tver.ru (A.I.S.); 2 A.N. Nesmeyanov Institute of Organoelement Compounds, Russian Academy of Sciences, 119991 Moscow, Russia; popov_a.y@mail.ru; 3 Regional Technological Centre, Tver State University, Zhelyabova Str., 33, 170100 Tver, Russia; 4 Ecole Polytechnique Fédérale de Lausanne, GGRC-ISIC-EPFL, CH-1015 Lausanne, Switzerland

**Keywords:** polymeric catalysts, hydrogenation of alkynols, palladium, hyper-cross-linked polystyrene, catalyst support porosity

## Abstract

This work is addressing the selective hydrogenation of alkynols over hybrid catalysts containing Pd-nanoparticles, within newly synthesized hyper-cross-linked polystyrenes (HPS). Alkynols containing C_5_, C_10_, and C_20_ with a terminal triple bond, which are structural analogues or direct semi-products of fragrant substances and fat-soluble vitamins, have been studied. Selective hydrogenation was carried out in a batch mode (ambient hydrogen pressure, at 90 °C, in toluene solvent), using hybrid Pd catalysts with low metal content (less than 0.2 wt.%). The microporous and mesoporous HPS were both synthesized and used as supports in order to address the influence of porosity. Synthesized catalysts were shown to be active and selective: in the case of C_5_, hydrogenation selectivity to the target product was more than 95%, at close to complete alkynol conversion. Mesoporous catalysts have shown some advantages in hydrogenation of long-chain alkynols.

## 1. Introduction

At present, palladium-catalyzed reactions can be considered as the most abundant in fine chemistry, which makes palladium catalysts extremely important [1,2,3,4,5,6,7]. Recently, there has been increased interest in environmentally benign Pd-catalyzed processes that are also economically viable. Indeed, the use of transition metals in organic synthesis is associated with some critical drawbacks, such as their high cost, toxicity, separation, and leaching of catalytically active forms of metals. Pd is considered to give a small environmental impact and is regarded as a relatively low-toxicity material. However, it was also shown that, in some forms and at high concentrations (e.g., in the form of PdCl_2_), Pd is toxic and carcinogenic [8,9,10]. The increased durability and reusability of Pd-catalysts strongly depends on the choice of suitable and stable support. It is noteworthy that the synthesis of nanoparticulate Pd-containing catalysts with low metal loading is of high importance, especially for the reactions, which require the well-defined Pd(0) surface [11], such as three-phase hydrogenation processes.

**Figure 1:**
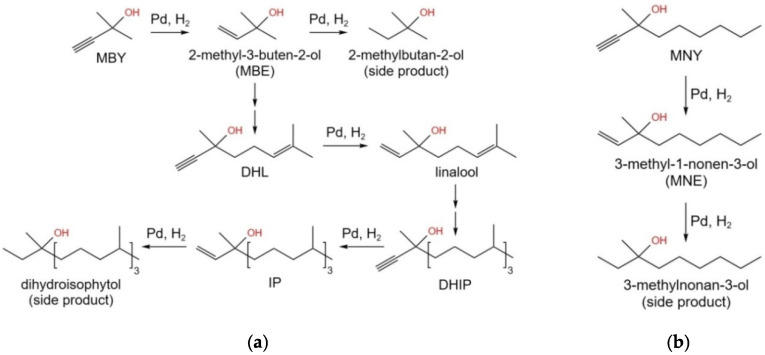
Simplified scheme of MBY transformation to isophytol (IP)—so-called “acetylenic process” (**a**) and scheme of MNY hydrogenation (**b**).

Polymeric supports have several advantages in comparison with traditional oxidic supports or carbon, i.e., they can be tailor-made for reactions of interest [12]. Porous polymers were synthesized about fifty years ago [13], by incorporating di-/multitopic monomers into well-known step-growth and chain-growth polymerization processes to provide cross-links between propagating polymer chains, yielding three-dimensional network materials. Until today, the most popular class of these materials is based on the polystyrene-divinylbenzene (PS-DVB) system [14]. Hyper-cross-linked polystyrene (HPS) networks and sorbents, described for the first time in the scientific literature in the early 1970s, came into routine practice by the end of the 1990s [15]. HPS possesses high sorption potential due to a specific structure that, in turn, is determined by the synthesis conditions [16]. In spite of a number of commercially available HPS resins, there is still room for the improvement of the synthesis procedure aiming at better catalytic properties, for the reaction of interest. This work describes the synthesis of HPS-based catalysts with Pd content below 0.2 wt.%, for the selective hydrogenation of alkynols to the corresponding olefinic alcohols at ambient hydrogen pressure. The following alkynols were studied (see Figure 1): 2-methyl-3-butyn-3-ol (MBY), 3-methyl-1-nonyn-3-ol (MNY) and dehydroisophytol (DHIP). MBY and DHIP are semiproducts in the synthesis of fat-soluble vitamins, such as A, E, and K [17,18,19], while MNY is a model compound, which closely relates to the very industrially important alkynol—3,7-dimethyl-6-octen-3-ol (dehydrolinalool, DHL) [20,21].

In our previous works [22,23,24,25], we have studied the catalytic properties of nanoparticulate Pd/HPS systems. It was shown that in the reaction of MBY semi-hydrogenation, more than 95% selectivity was achieved at complete substrate conversion, while using commercial non-functionalized HPS as a support. MBY (C_5_alkynol) is a substrate with relatively low molecular weight and can be successfully hydrogenated in the presence of Pd nanoparticles (NPs) confined within a highly porous polymeric network. For terminal alkynols with higher chain length, such as DHL (C_10_) or DHIP (C_20_), the porous structure may lead to noticeable internal-diffusion limitations affecting catalytic efficiency (activity/selectivity). Thus, in this work we explore, for the first time, the influence of HPS porosity on the catalytic properties of Pd-containing polymers.

## 2. Results

Four samples of HPS were synthesized using different porogenes: alcohol C7, polystyrene (PS), and oil fractions (OF2 and OF3). HPS samples were characterized by the method of low-temperature nitrogen physisorption, X-ray photoelectron spectroscopy (*XPS*), X-ray Fluorescence Analysis (*XFA*), diffuse reflectance infrared spectroscopy (*DRIFT*), and scanning electron microscopy (*SEM*).

**Figure 2:**
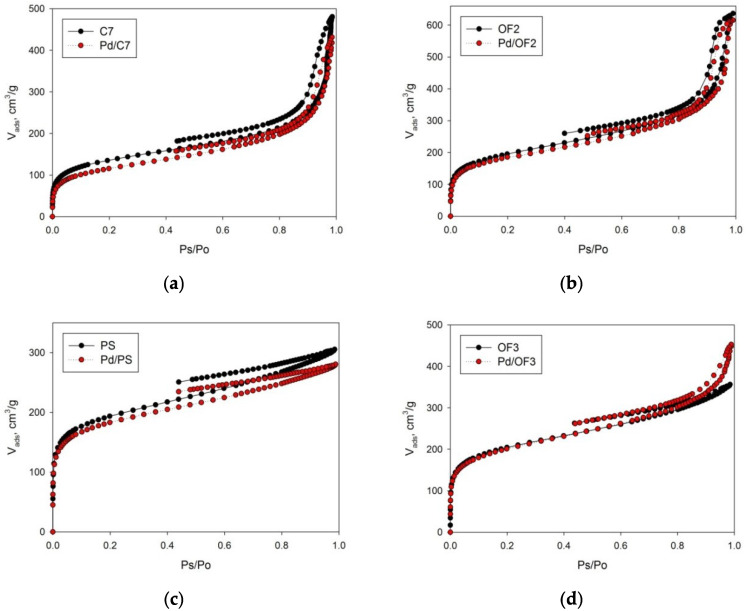
Adsorption–desorption isotherms: (**a**) C7 and Pd/C7; (**b**) OF2 and Pd/OF2; (**c**) PS and Pd/PS; (**d**) OF3 and Pd/OF3.

**Figure 3:**
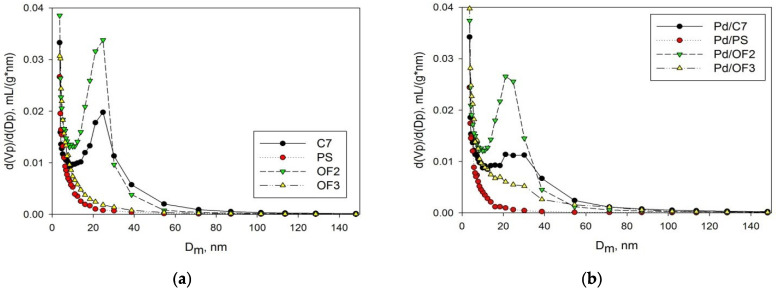
Comparison of pore-volume distributions of synthesized HPS samples (**a**) and Pd/HPS catalysts (**b**).

The results of low-temperature nitrogen physisorption for all samples are presented in Figure 2 and Figure 3. As can be seen, two samples, C7 and OF2, have mesoporous structure, while the others (PS and OF3) have predominant microporosity [26]. The values of the specific surface area (SSA) of C7 and OF2 are 490 ± 10 m^2^/g and 700 ± 14 m^2^/g (calculated according to the BET model), respectively. The SSA values of PS and OF3 are 680 ± 14 m^2^/g and 720 ± 14 m^2^/g, respectively. The following values of the micropore volume were found: 0.12 mL/g (C7), 0.15 mL/g (OF2), 0.18 mL/g (PS), and 0.20 mL/g (OF3). The indicated values comprise 15.8%, 15.5%, 37.5%, and 37.2% of the total pore volume of each sample, respectively. Synthesized polymers were used as supports for Pd deposition by the wet-impregnation method (see Section 3). Catalysts were designated as Pd/C7 (0.11 wt.% Pd, according to the data of *XFA*), Pd/PS (0.16 wt.% Pd), PdOF2 (0.11 wt.% Pd), and Pd/OF3 (0.17 wt.% Pd). Low-temperature nitrogen physisorption (see Figure 2 and Figure 3 and Table S1) revealed that after Pd deposition, SSA and porosity have not changed significantly. Mean diameter of mesopores in the Pd/C7 and Pd/OF2 catalysts remains unchanged (about 25 nm), for the initial polymer supports. So, we conclude that the observed differences in BET SSA and porosity after impregnation are negligible, which is logical due to the very low amount of Pd deposited (<0.2 wt.%). Note that all these values were obtained ex situ for the dry polymeric networks and catalysts, while the swelling occurs in situ under hydrogenation conditions in toluene medium, which additionally smooths the observed differences. However, the general trend of porosity influence can be revealed while comparing “microporous” and “mesoporous” samples.

**Figure 4:**
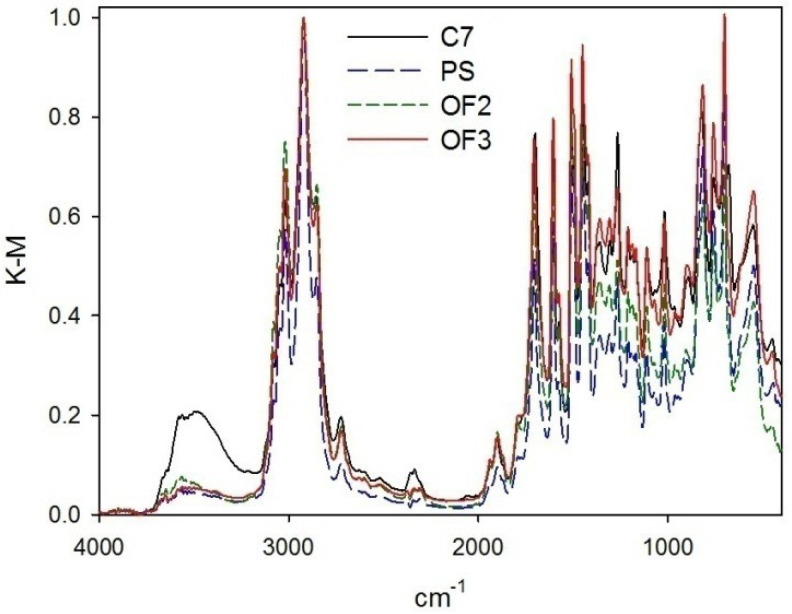
Normalized *DRIFT* spectra of synthesized polymers.

*DRIFT* spectra of the synthesized polymers are presented in Figure 4. It is noteworthy that all the spectra were normalized; the chosen band at 2922 cm^−1^ corresponds to the stretching vibrations of the alkanes (ν_C-H_).

For all the HPS samples, the following main adsorption bands were found: ν_C-H_ of alkanes (2922 cm^−1^ and 2858 cm^−1^) and δ_C-H_ of alkanes (1450 cm^−1^ and 1421 cm^−1^), ν_C-H_ of benzene rings (3020 cm^−1^), ν_C=O_ in CO_2_ (about 2357 cm^−1^ and 2337 cm^−1^), ν_C=O_ of carbonyl carbon (1699–1707 cm^−1^), ν_C-C_ of benzene rings (1605 cm^−1^ and 1508 cm^−1^), and carboxyl ν_–OH_ in carboxylic groups (a broad asymmetric peak in the range of 3500–2500 cm^−1^), ν_C–O_ in carboxyl groups (1306 cm^−1^), in-plane vibrations δ_C-H_ of benzene rings in the range of 1250–950 cm^−1^ (of no practical importance), and out-of-plane vibrations δ_C–H_ of benzene rings in the range of 900–650 cm^−1^ (characterizing mono-, di-, and trisubstituted benzene rings).

C7 is the sole only sample, which has obvious differences in the *DRIFT* spectrum, i.e., higher intensity of the band corresponding to ν_–OH_ in the hydroxyl groups (3650 cm^−1^ and 3570 cm^−1^), which can be due to alcohol utilized as porogen.

**Figure 5:**
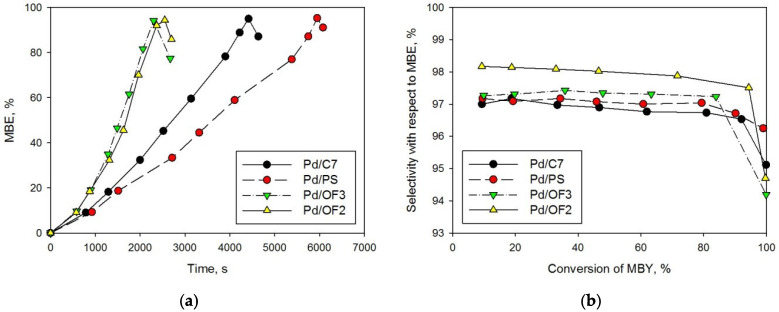
Kinetic curves of 2-methyl-3-buten-2-ol (MBE) accumulation (**a**) and dependence of the selectivity with respect to MBE on MBY conversion of (**b**).

HPS-based Pd-containing catalysts were tested in the reaction of the selective hydrogenation of MBY in a batch mode at 90 °C, with toluene as a solvent. It was found that Pd/OF2 and Pd/OF3 have the highest activity (Figure 5a), while the selectivity for all samples was similar (96–97% at 95% of MBY conversion, see Figure 5b and Table 1, #1–4).

The difference in the observed activity has no dependence on the samples’ porosity and can be due to the different distribution and sizes of Pd NPs. It is noteworthy that all the initial samples were unreduced and contained Pd(II). As it was supposed in one of our previous publications [27] and also shown in the work of Hu K.-J. et al. [28], formation of Pd NPs occurs effectively in situ in the presence of the substrate. A slight induction period, which can be seen on the presented kinetic curves (Figure 5a), confirms that preliminary treatment of the catalysts with molecular hydrogen in a liquid phase (toluene) at 90 °C, ambient pressure did not allow for complete Pd reduction, and the formation of active centers continued in situ under reaction conditions.

**Figure 6:**
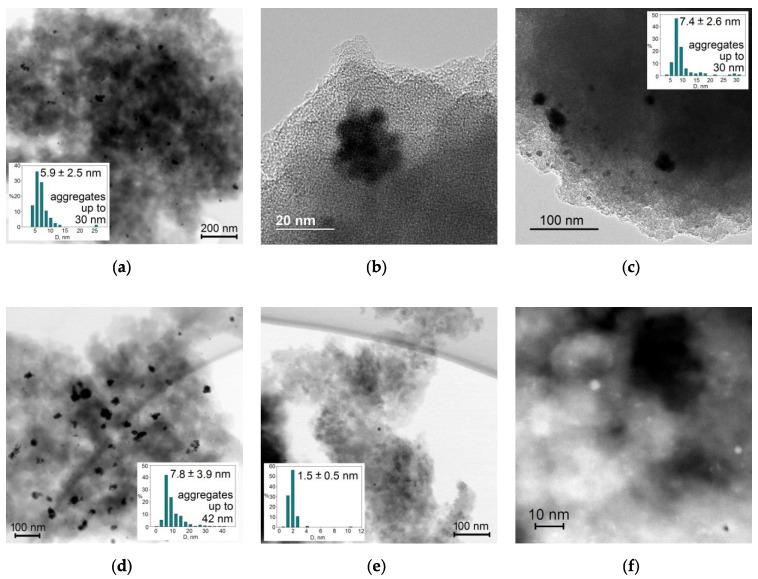
S/TEM images of catalysts taken after the reaction of MBY hydrogenation: (**a**) Pd/C7 (scale 100 nm), (**b**) Pd/C7 (scale 20 nm), (**c**) Pd/PS (scale 100 nm), (**d**) Pd/OF3 (scale 100 nm), (**e**) Pd/OF2 (scale 100 nm), and (**f**) HAADF STEM of Pd/OF2 (scale 10 nm).

After the MBY hydrogenation, the catalysts were separated by vacuum filtration, thoroughly washed with toluene and chloroform, dried at 65 °C overnight, and the Pd NPs’ sizes were analyzed by scanning/transmission electron microscopy (S/TEM) (Figure 6).

It was found that among these four samples, Pd NPs were more evenly distributed in Pd/OF2 (Figure 6e,f) and Pd/C7 (Figure 6a,b), which were synthesized using micro-mesoporous polymers. NPs with the lowest mean diameter, of 1.5 ± 0.5 nm, and an absence of aggregates were found in Pd/OF2 (Figure 6e,f), where only a few particles with a diameter about 10 nm were observed. In the case of Pd/C7, the mean diameter of Pd NPs was 5.9 ± 2.0 nm (Figure 6a). Moreover, these NPs formed aggregates up to 30 nm in diameter (Figure 6b), likely due to their migration after nucleation under reaction conditions. For other two samples, the mean diameters of Pd NPs were 7.4 ± 2.6 nm (Pd/PS, Figure 6c) and 7.8 ± 3.9 nm (Pd/OF3, Figure 6d). Moreover, a number of dense aggregates with diameters up to 42 nm were found, especially in the case of Pd/OF3. The formation of larger Pd NPs and their aggregates cannot be explained by the about 1.5-fold higher Pd amount for these two samples (Pd/PS and Pd/OF3), in comparison with the other ones (Pd/C7 and Pd/OF2). In our previous study, using commercial HPS with predominant microporosity and impregnated by Pd acetate, we found that irrespective of palladium loading in a relatively wide range (0.5–2.0 wt.%) and reduction method, NPs were evenly distributed and had diameters of 2–5 nm [22,23].

**Figure 7:**
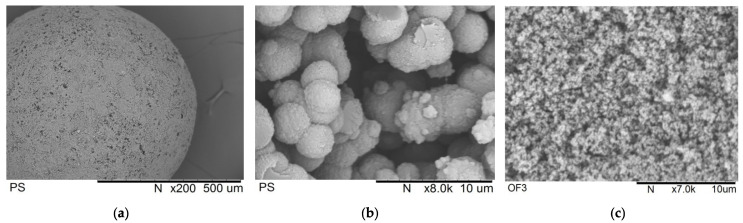
*SEM* images of PS and OF3: (**a**) initial PS granule (scale 500 µm), (**b**) crushed PS (scale 10 µm), and (**c**) crushed OF3 (scale 10 µm).

It is noteworthy that PS and OF3 also had gigue pores, which cannot be measured by the low-temperature nitrogen physisorption but were clearly identified in the SEM images (Figure 7). Such huge pores, which are visible not only on the outer surface of the initial polymer granules (Figure 7a) but also in their internal structure (Figure 7b,c), facilitate mass transport of metal precursor. Thus, a lot of Pd aggregates can be found closer to the outer surface of the catalysts taken after hydrogenation experiments.

Obviously, there is no direct correlation between the observed sizes of Pd NPs and the catalytic activity of HPS-based samples in the reaction of MBY hydrogenation, since the NPs formation and possible aggregation occurred during preliminarily liquid-phase reduction and also in situ. It is known [29] that for relatively big particles (about 6–13 nm) the rate of MBY hydrogenation is almost independent of the particle sizes. In the absence of aggregation, smaller Pd NPs, with sizes less than 2 nm, usually possesses lower activity in hydrogenation reactions of unsaturated compounds [30]. However, small NPs are proven to have higher selectivity [31,32]. As can be seen (Figure 5b), Pd/OF2 revealed the highest selectivity (about 98% until 80% conversion of MBY) among all the samples. The highest activity of Pd/OF2 (Table 1, #3) can be attributed to the absence of Pd NPs aggregates, while the activity of all other samples suffers from noticeable aggregation of palladium NPs during the reaction.

**Figure 8:**
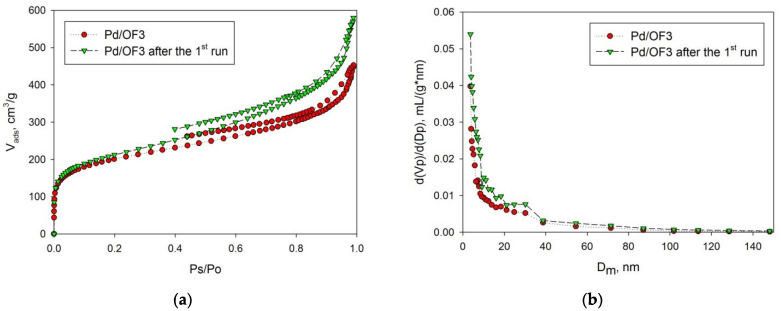
Comparison of adsorption–desorption isotherms (**a**) and pore-volume distributions (**b**) for Pd/OF3: initial and taken after the MBY hydrogenation.

Catalyst Pd/OF3 was studied by the method of low-temperature nitrogen physisorption, after the reaction of MBY hydrogenation (Figure 8). It was found that BET SSA slightly increased after the reaction from 710 ± 14 m2/g up to 750 ± 15 m2/g. This increase in SSA was accompanied by decrease in micropore volume from 0.19 mL/g to 0.12 mL/g(that comprised nearly 13% of the total pore volume). The observed changes in porosity are likely due to the nucleation of Pd NPs followed by redistribution and agglomeration during the reaction.

Samples Pd/OF2 and Pd/OF3 were characterized by the *XPS* method. It was shown that in both initial catalysts, Pd acetate on the surface was transformed to PdO(binding energy (BE) of Pd 3d_5/2_ was equal to 335.7–335.6 eV [33]) during the catalysts’ impregnation and subsequent storage. It is noteworthy that Pd content on the surface was very low (0.03–0.05 at.%),which did not allow reliable quantitative estimation. After the MBY hydrogenation, Pd content on the surface (in at.%) remained nearly the same; the results of *XFA* additionally revealed that no Pd loss occurred during the reaction in toluene medium. It is interesting that the BE of Pd 3d_5/2_ was 337.1 eV in both used samples, which is lower compared to the initial catalysts and may indicate the formation of larger NPs of PdO (note that after the reaction the samples were filtrated, dried, and stored under air, which resulted in the oxidation of Pd NPs).

**Figure 9:**
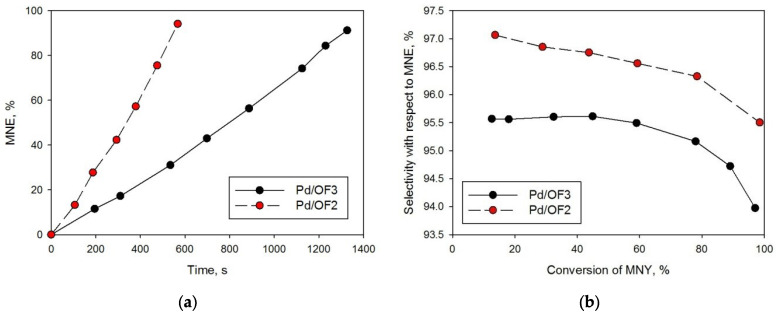
Kinetic curves of 3-methyl-1-nonen-3-ol (MNE) accumulation (**a**) and dependence of the selectivity, with respect to MNE on MNY conversion of (**b**).

**Figure 10:**
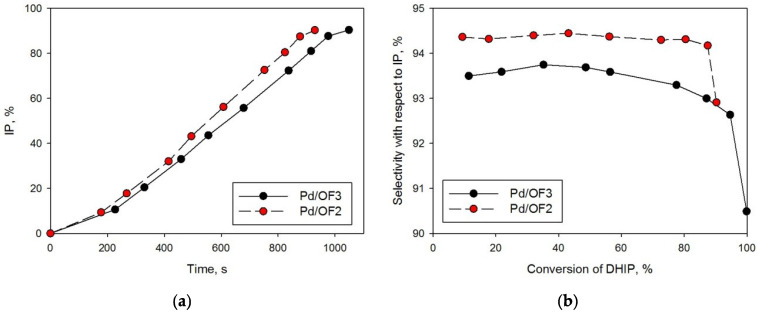
Kinetic curves of IP accumulation (**a**) and dependence of the selectivity, in respect to IP on DHIP conversion of (**b**).

Pd/OF2 and Pd/OF3, which have the highest weight-swelling ratio in toluene (see Section 4.2) were chosen for hydrogenation of MNY (Figure 9, Table 1, #5–6) and DHIP (Figure 10, Table 1, #7–8). In contrast to MBY, in the case of alkynols C_10_ (MNY) and C_20_ (DHIP), the catalytic activity related to the palladium content was noticeably higher for the mesoporous sample (Pd/OF2) in comparison with the microporous one (Pd/OF3).

The difference in activity between Pd/OF2 and Pd/OF3 is mostly noticeable in the hydrogenation of MNY. In order to verify if pore sizes influence the observed hydrogenation rates, unrestricted DFT calculations were carried out for MBY, MNY, and DHIP at the BP level of theory using triple-zeta basis sets (see Figure S1 in the Supporting Materials).

It was found (see Figures S1–S3) that MBY molecules have a collision size (5.91 × 5.93 × 7.15 Å) that is much smaller than the pore diameters. The collision size of MNY is 5.95 × 6.23 × 14.16 Å. The collision size of DHIP is 6.09 × 8.30 × 21.99 Å. Obviously, for Pd/PS and Pd/OF3 catalysts, MNY and DHIP will undergo strong steric hindrances in micropores (below 2 nm) and in small mesopores (2–6 nm), which will cause internal diffusion limitations. At the same time, for the sample Pd/OF3 there is almost no difference in the hydrogenation rates of MBY and MNY (Table 1, #4, and #6). However, in the case of DHIP (Table 1, #8), in spite of the obvious steric hindrances, the hydrogenation rate is 1.8 times higher in comparison with MNY (Table 1 and #6). The higher hydrogenation rate is likely due to the positive inductive effect of the alkyl chain to the triple bond, which makes it more reactive. Relatively lower selectivity, with respect to olefinic alcohol, in the case of DHIP (see Table 1 and #8), in comparison with other alkynols with a shorter alkane chain (Table 1, #6, and #4), can be due to the higher hydrophobicity of DHIP (calculated dipole moment (µ) of DHIP is 1.05 D, µ(MNY) = 1.22 D, µ(MBY) = 1.24 D), which results in its higher affinity to the polymeric HPS support.

It can be proposed that the mesoporous structure along with the small sizes of Pd NPs (about 2 nm) are beneficial for the hydrogenating substrates with short alkane chains. For high-molecular weight alkynols, such as DHIP, the existence of mesopores (sample Pd/OF2) results in another effect—the blockage of pores, presumably by hydrogenation products. As a result, an incomplete conversion of DHIP (90%) and a fast decrease in selectivity, with respect to IP at high conversions, were found while using Pd/OF2 (Figure 9b, Table 1, and #7).

## 3. Discussion

The results reported here demonstrate that palladium NPs confined in a polymeric network of HPS can catalyze the selective hydrogenation of alkynols C_5_, C_10_, and C_20_. HPS supports with different porosity were synthesized and divided into two groups: (i) “microporous” (PS and OF3), with the share of micropores about 37% by volume and also numerous giguepores, as confirmed by *SEM* study; and (ii) “mesoporous” (C7 and OF2), with the share of micropores about 15% by volume and the presence of mesopores with a mean diameter about 25 nm.

HPS-based catalysts with low Pd loadings (0.11–0.17 wt.%) have been prepared via the wet-impregnation of the polymers with Pd acetate. It was shown that impregnation has a negligible effect on polymer porosity, although slight blockage of the micropores takes place. Moreover, by the example of Pd/OF2 and Pd/OF3, it was shown that during the impregnation procedure the palladium acetate was decomposed to PdO (BE is 337.5–337.6 eV, according to the XPS data). All the catalysts were reduced in situ under the reaction conditions and tested in hydrogenation of alkynols—MBY, MNY, and DHIP. In the hydrogenation of MBY, selectivity, with respect to MBE, was about 95% at complete substrate conversion, which is typical for HPS-based catalysts [22,23]. The catalytic activity, characterized by the initial rate of MBY transformation related to Pd content, was remarkably high for two samples—Pd/OF2 and Pd/OF3: 25.4 mol_sub_/(mol_Pd_*s) and 17.2 mol_sub_/(mol_Pd_*s), respectively. For comparison, in the case of commercial Lindlar catalyst (2%-Pd/CaCO_3_) *R*_0_ = 3.3 mol_sub_/(mol_Pd_*s) under the same reaction conditions (90 °C, toluene).In the case of MBY, the influence of HPS porosity on the catalytic performance seems to be negligible, and the most important factor is the small size of Pd NPs. However, formation in situ of small NPs depends on the support morphology. Thus, the giguepores in the OF3structure may provide intensive NPs migration, resulting in aggregates and the diminished active surface of Pd.

While comparing two samples (Pd/OF2 and Pd/OF3) in the hydrogenation of long-chain alkynols (MNY and DHIP), it was shown that Pd/OF2 having mesopores and tiny Pd NPs (about 1.5 nm) is highly active in the hydrogenation of MNY (C_10_alkynol), while for DHIP (C_20_alkynol) the steric hindrance results in a decrease in catalytic activity and, likely, blockage of pores by both substrate and products. The sample Pd/OF3 presents close values of catalytic activity (*R*_0_) for MBY and MNY. It is noteworthy that Pd/OF3 reveals higher activity for DHIP in comparison with MNY, which could be due to the higher reactivity of triple bond of DHIP and its relatively low dipole moment that is responsible for high affinity to HPS.

However, the existence of giguepores with intensive mass-transfer results in aggregation of Pd NPs that makes OF3 less suitable for support, in comparison with “mesoporous” OF2. Thus, the influence of the support porosity is two-fold: (i) for the stage of catalyst preparation and formation of Pd NPs, it controls the distribution of catalytically active phase, sizes of NPs, and their tendency to aggregate; (ii) during the catalytic reaction, support porosity influences the accessibility of active sites to the reactants, but this factor can be responsible for the observed activity only if Pd NPs are equal in size. The complexity of the catalytic systems and the multiplicity of the interrelated parameters do not allow to determine which is the crucial one (see Table S1), but the general trend is as follows: mesoporous polymeric materials with high swelling ability (i.e., OF2) allow for more even distribution of the catalytically active phase that provides excellent activity, and for such systems the length of the alkane chain matters (due to different induction effect, hydrophobicity, and diffusion limitations). In contrast, when Pd aggregates are formed as a result of the NPs migration into the huge pores of some polymers (present along with micropores, such asin OF3), the interpretation of porosity influence on catalyst performance becomes hectic.

## 4. Materials and Methods

### 4.1. Materials

The 2-Methyl-3-butyn-2-ol (MBY, 99%) was purchased from Fluka (Buchs, Switzerland). The 1-Methyl-3-nonyn-1-ol (MNY, 97%) and methanol (99%) were purchased from Alfa Aesar (Ward Hill, MA, USA). Dehydroisophytol (DHIP, 97%) was purchased from DSM (Heerlen, The Netherlands). Divinylbenzene (DVB, 80%), tetrahydrofuran (THF, ≥99.9%), toluene (≥99.5%), hexane (≥99%), and chloroform (≥99%) were obtained from Sigma-Aldrich (St. Louis, MO, USA). Styrene (STY, ≥99%), FeCl_3_ (≥99%), and benzoyl peroxide (BPO, 75%) were purchased from Across Organics. Polyvinyl alcohol (GM-14) was obtained from Nippon Synthetic. The 1,2-Ethylene was purchased from “Ufareaktiv” (Ufa, Russia). K_2_Cr_2_O_7_, NaCl, paraform powder were purchased from “Reakhim” (Moscow, Russia). Polystyrene (PSt, GPP #585, Mw = 3.5 × 10^5^ g/mol) JSC “Nizhnekamskneftekhim” (Nizhnekamsk, Russia). Petroleum solvent (OF, oil fraction “nefras—S”; boiling temperature 100–170 °C;) was obtained from “Himproduktbalahna” (Gidrotorf, Russia). Palladium acetate (Pd(CH_3_COO)_2_, Pd content 47.68%) was purchased from JSC “Aurat” (Moscow, Russia). Reagent-grade hydrogen of 99.999% purity was received from AGA. All chemicals were used as received, except for 1,2-ethylene dichloride, which was purified by distillation over P_2_O_5_. Distilled water was purified with an ELSI–AQUA water purification system.

### 4.2. Synthesis and Characterization of Polymeric Supports

All initial STY-DVB copolymers were synthesized by a free radical copolymerization of monomers, by the suspension method. In a typical experiment, 180 mL of aqueous phase and 40 mL of organic phase were placed in three-necked round-bottom flask equipped with a reflux condenser, thermometer, and mechanical stirrer. The synthesis was carried out at 80 °C for 8 h, with agitation. Obtained polymer beads were filtered and rigorously washed with hot water, acetone, and r.t. water, and then dried in air at 60–80 °C.

The aqueous phase contained 0.94 g of polyvinyl alcohol GM-14 as the suspension stabilizing surfactant, 9.04 g of NaCl, 0.50 g of K_2_Cr_2_O_7_, and 180 mL of water. The organic phases comprised of STY, DVB, 2 wt.%BPO, and porogen at a monomer/porogen volumetric ratio of 1.5–3 *v*/*v* (see Table 2).

Samples of the HPS series were synthesized by the cross-linking of the copolymers with monochlorodimethylether using 1 mole of ether to STY unit, according to the procedure described elsewhere [34,35]. The degree of cross-linking estimates was 200%. Resulting polymers were designated as C7, PS, OF2, and OF3.

For all the synthesized polymers, the weight–swelling ratio (*Q_w_*) in toluene (the solvent used for hydrogenation reactions) was found, according to the procedure described elsewhere [34]. In a typical measurement, about 1.5 mL of polymer swollen in toluene was placed in a porous-bottom tube, which was then sealed; the interbead liquid was removed by centrifugation at 3000 rpm for 5 min. The polymer was then transferred to a preliminarily weighed sample bottle, and the bottle with the polymer was weighed. After that, the sorbent was dried in an oven at 110 °C to a constant weight. The weight–swelling ratio was calculated as the amount of the solvent (mL) retained by one gram of the dry polymer. Thus, the following values of *Q_w_* were found: 1.18 mL/g (C7); 1.23 mL/g (PS); 1.64 mL/g (OF2); and 1.57 mL/g (OF3).

Morphology of the samples PS and OF3 was additionally characterized by Scanning Electron Microscopy (*SEM*) using a TM3000 instrument (Hitachi, Japan). The samples were glued onto double-sided electrically conductive carbon tape and were treated by vacuum spraying of gold. The scanning voltage was 30 kV.

Moreover, pretreated (washed, dried, and crushed; see Section 4.3) polymers were characterized by liquid nitrogen physisorption and Diffuse Reflectance Infrared Fourier Transform Spectroscopy (*DRIFTS*).

*Liquid nitrogen physisorption* was carried out to determine SSA and porosity using a Beckman Coulter SA 3100 (Coulter Corporation, Brea, CA, USA). Prior to the analysis, each sample was placed in a quartz cell installed in the Beckman Coulter SA-PREP. The samples were pretreated over 60 min under nitrogen at 120 °C. Once the pretreatment was complete, the cell was cooled, weighed, and then transferred to the analytical port. Analysis was performed at −196 °C and a relative pressure of 0.9814 (for pores less than 100 nm in diameter) to obtain a PSD (ADS) profile. The accuracy of the SSA measurement for the BET model is 2%.

*DRIFTS* was carried out using an IRPrestige-21 FTIR spectrometer (Shimadzu, Japan) equipped with a DRS-8000 diffuse reflectance accessory (Shimadzu, Japan). The background sample was a mirror of the material of the optical system of the DRS-8000 accessory. All spectra were recorded in the 4000–500 cm^−1^ range of wavenumbers at a resolution of 4 cm^−1^.

### 4.3. Synthesis of Catalysts

Pd-containing HPS-based catalysts were synthesized via wet-impregnation. In a typical experiment, 0.5 g of pretreated (washed with hexane at 60 °C, dried at 65 °C for 5 h, and crushed) granules of HPS were impregnated with 1.2 mL of the THF solution of the precursor (Pd(CH_3_COO)_2_) of a chosen concentration (0.0197 mol/L). The HPS samples impregnated with Pd were dried at 65 °C, until the constant weight was achieved.

Thus, the catalysts Pd/C7, Pd/PS, Pd/OF2, and Pd/OF3 were synthesized containing 0.11 wt.%, 0.16 wt.%, 0.11 wt.%, and 0.17 wt.% of palladium, respectively (according to the data of *XFA*).

### 4.4. Reaction Procedure and Analysis of Reaction Mixture

Testing of Pd-containing HPS-based samples in hydrogenation of alkynols was carried out in toluene medium at 90 °C and ambient hydrogen pressure in a 60 mL isothermal glass-batch reactor installed in a shaker at vigorous stirring (more than 800 two-sided sharing per minute). The total volume of the liquid phase was 30 mL. A recirculating bath (LOIP LT 100, Saint-Petersburg, Russia) was used to stabilize the reaction temperature within ±1 °C, with water as a heating medium. The reactor was connected to a gasometrical burette for *on-line* hydrogen consumption control. At the beginning of each experiment the temperature was set and allowed to stabilize, so the reactor was charged with the catalyst, and hydrogen was then introduced and the catalyst was kept under hydrogen for 60min. Then, substrate was added and the reaction started.

Samples of the reaction mixture were analyzed via GC (Kristallux 4000M, Yoshkar-Ola, Russia) equipped with FID and capillary column ZB-WAX (60 m × 0.53 mm i.d., 1 μm film thickness). Helium was used as a carrier gas. The concentrations of the reaction mixture components were calculated using the absolute *calibration* method using chemically pure components.

Conversion of substrates was defined as:

*X* (%) = (*C_sub,_*_0_ − *C_sub,i_*) × *C_sub,0_*^−1^ × 100. (1)

Selectivity with respect to the target product was given as:

*S* (%) = *C_prod,i_* × (*C_sub,_*_0_ − *C_sub,i_*)^−1^ × 100. (2)

Reaction rate was designated as *R*_0_, [mol_sub_∙mol_Me_^−1^∙s^−1^]:

*R*_0_ = (*C_sub,_*_0_ − *C_sub,i_*) × (*C_Me_* × *τ_i_*)^−1^. (3)

It is noteworthy that *R*_0_ was calculated in the range of concentrations, corresponding to the linear part of the kinetic curves of alkynols’ conversion vs. time (induction period was excluded).

### 4.5. Catalyst-Characterization Methods

HPS-based catalysts were characterized by te liquid nitrogen physisorption, X-ray Fluorescence Analysis (*XFA*), X-ray photoelectron spectroscopy (*XPS*), and Scanning Transmission Electron Microscopy (*S/TEM*).

*Liquid nitrogen physisorption* was carried out to determine SSA and porosity, according to the procedure described above (Section 4.2).

*XFA* was carried out to determine the Pd content. It was performed with a VRA-30 spectrometer (Carl Zeiss, Jena, Germany) equipped with an Mo anode, LiF crystal analyzer, and SZ detector. Analyses were based on the Co Kα line, and a series of standards prepared by mixing 1 g of catalyst with 10–20 mg of standard compounds. The time of data acquisition was constant at 10 s.

*XPS* data were obtained using Mg Kα (hν = 1253.6 eV) radiation with an ES-2403 spectrometer (Institute for Analytic Instrumentation of RAS, Saint Petersburg, Russia) equipped with an energy analyzer, PHOIBOS 100-MCD5 (SPECS, Berlin, Germany) and X-ray source XR-50 (SPECS, Berlin, Germany). All the data were acquired at an X-ray power of 250 W. Survey spectra were recorded at an energy step of 0.5 eV with an analyzer pass energy of 40 eV. High-resolution spectra were recorded at an energy step of 0.05 eV with an analyzer pass energy of 7 eV. Samples were outgassed for 180 min before analysis and were stable during the examination. The data analysis was performed via CasaXPS. Binding energies (BEs) were determined with the error ±0.1 eV.

*S/TEM* characterization was carried out using an FEI TecnaiOsiris instrument(Thermo Fisher Scientific, Madison, WI, USA) operating at an accelerating voltage of 200 kV, equipped with a high-angle annular dark field (*HAADF*) detector (Fischione, Export, PA, USA) and an energy-dispersive X-ray (*EDX*) microanalysis spectrometer (EDAX, Mahwah, NJ, USA). Samples were prepared by embedding the catalyst in epoxy resin with following microtomming (*ca*. 50 nm thick) at ambient temperature. For the image processing Digital Micrograph (Gatan, Warrendale, PA, USA) software and a TIA (Thermo Fisher Scientific, Madison, WI, USA) were used. Holey carbon/Cu grid was used as a sample support.

## Figures and Tables

**Table 1:** Data of HPS-based catalysts, testing in hydrogenation ofalkynols (solvent—toluene, temperature 90 °C, ambient hydrogen pressure).

N	Catalyst	Substrate	Product	S_95_ ± 0.5, % ^2^	X_max_ (S) ± 0.5, % ^1^	*R*_0_ ± 0.1, mol_sub_/(mol_Pd_*s) ^3^
1	Pd/C7	MBY	MBE	96.0	99.9 (95.1)	10.2
2	Pd/PS			96.5	99.0 (96.3)	4.6
3	Pd/OF2			97.2	99.6 (94.7)	25.4
4	Pd/OF3			95.1	99.9 (94.2)	17.2
5	Pd/OF2	MNY	MNE	95.6	98.5 (95.5)	61.8
6	Pd/OF3			94.2	97.1 (94.0)	16.8
7	Pd/OF2	DHIP	IP	-	90.3 (92.9)	48.4
8	Pd/OF3			92.5	100 (90.5)	30.4

^1^ X_max_—maximum conversion observed in one experiment; ^2^ S_95_—selectivity calculated at X = 95%; ^3^ *R*_0_—initial transformation rate.

**Table 2:** The composition of the organic phases.

Initial Copolymer Designation	STY/DVB Molar Ratio	Porogen	Monomer/Porogen, *v*/*v*
C7	93/7	C_7_H_15_OH	1.5
PS	93/7	5% PSt solution in toluene	1.5
OF2	97/3	OF	2.0
OF3	95/5	OF	3.0

## Data Availability

Not applicable.

## Supplementary Materials

The following supporting information can be downloaded at: https://www.mdpi.com/article/10.3390/molecules27123842/s1. Figure S1: DFT calculation for MBY; Figure S2: DFT calculation for MNY; Figure S3: DFT calculation for DHIP; Table S1: Summary data on the physicochemical and catalytic properties of Pd/HPS.

## Sample Availability

Samples of the HPS-based catalysts are available from the authors.

## Abbreviations

BE, binding energy; BET, Brunauer–Emmett–Teller; DHIP, dehydroisophytol; DHL, dehydrolinalool; DRIFT, diffuse reflectance infrared spectroscopy; HPS, hyper-cross-linked polystyrene; IP, isophytol; MBE, 2-methyl-3-butene-2-ol; MBY, 2-methyl-3-butyn-3-ol; MNE,3-methyl-1-nonen-3-ol; MNY, 3-methyl-1-nonyn-3-ol; NP, nanoparticle; OF, oil fraction; PS, polystyrene; PS-DVB, polystyrene-divinylbenzene; SSA, specific surface area; STY, styrene; S/TEM, scanning transmission electron microscopy.
