# Supplementary material for: Hybrid Pd-Nanoparticles within Polymeric Network in Selective Hydrogenation of Alkynols: Influence of Support Porosity
Source: Molecules. 2022 Jun 15;27(12):3842. doi: 10.3390/molecules27123842 (PMC9228706; doi:10.3390/molecules27123842)
Supplement: Supplementary file 1 [file molecules-27-03842-s001.zip › molecules-1754878-supplementary.pdf]

SupplementaryMaterials

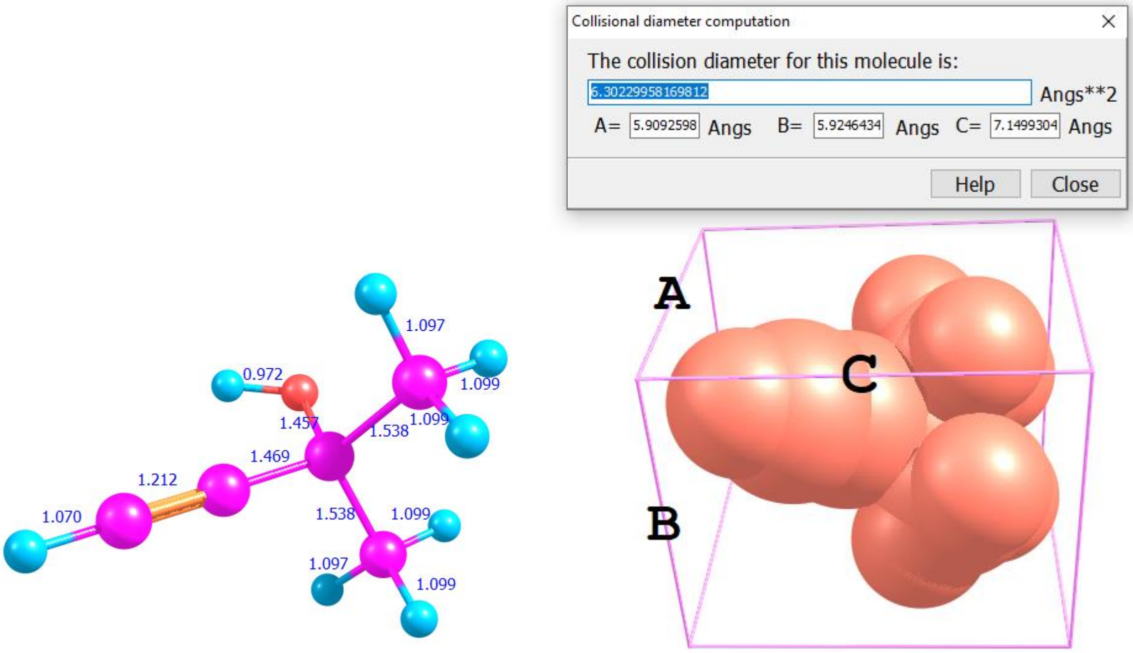

Figure S1.DFT calculation of MBY.

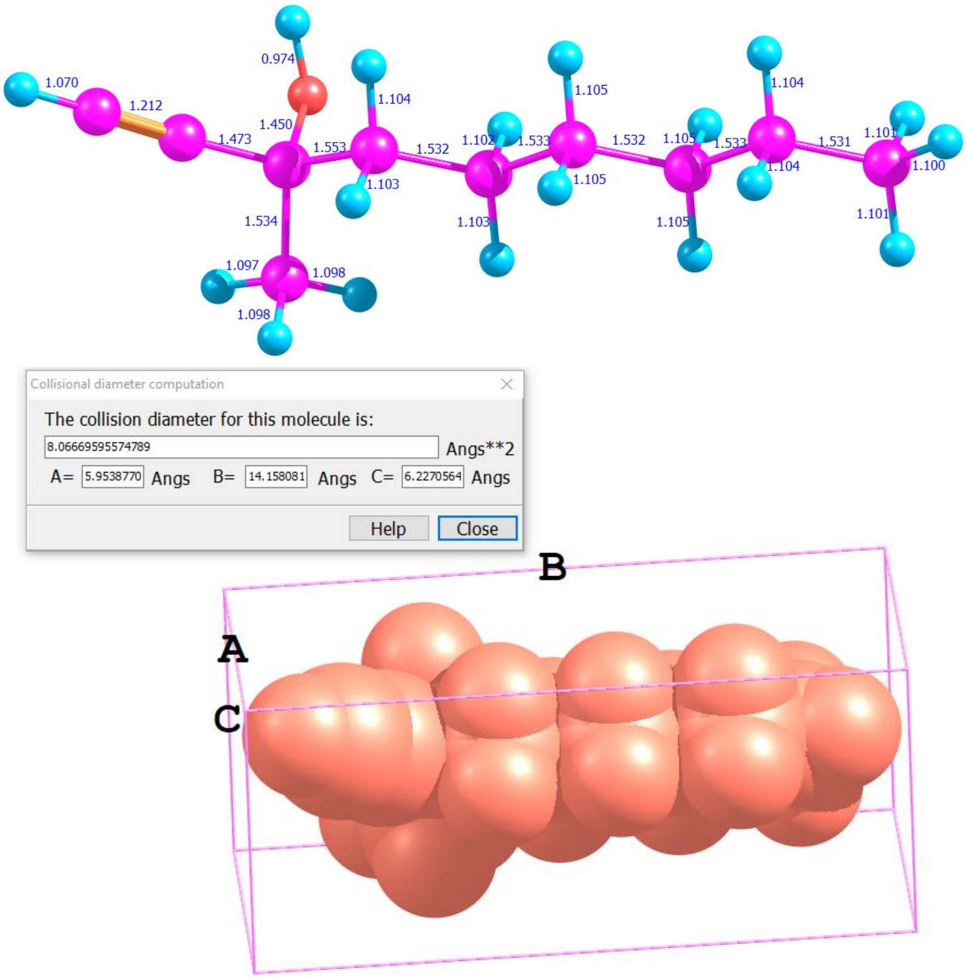

Figure S2.DFT calculation of MNY.

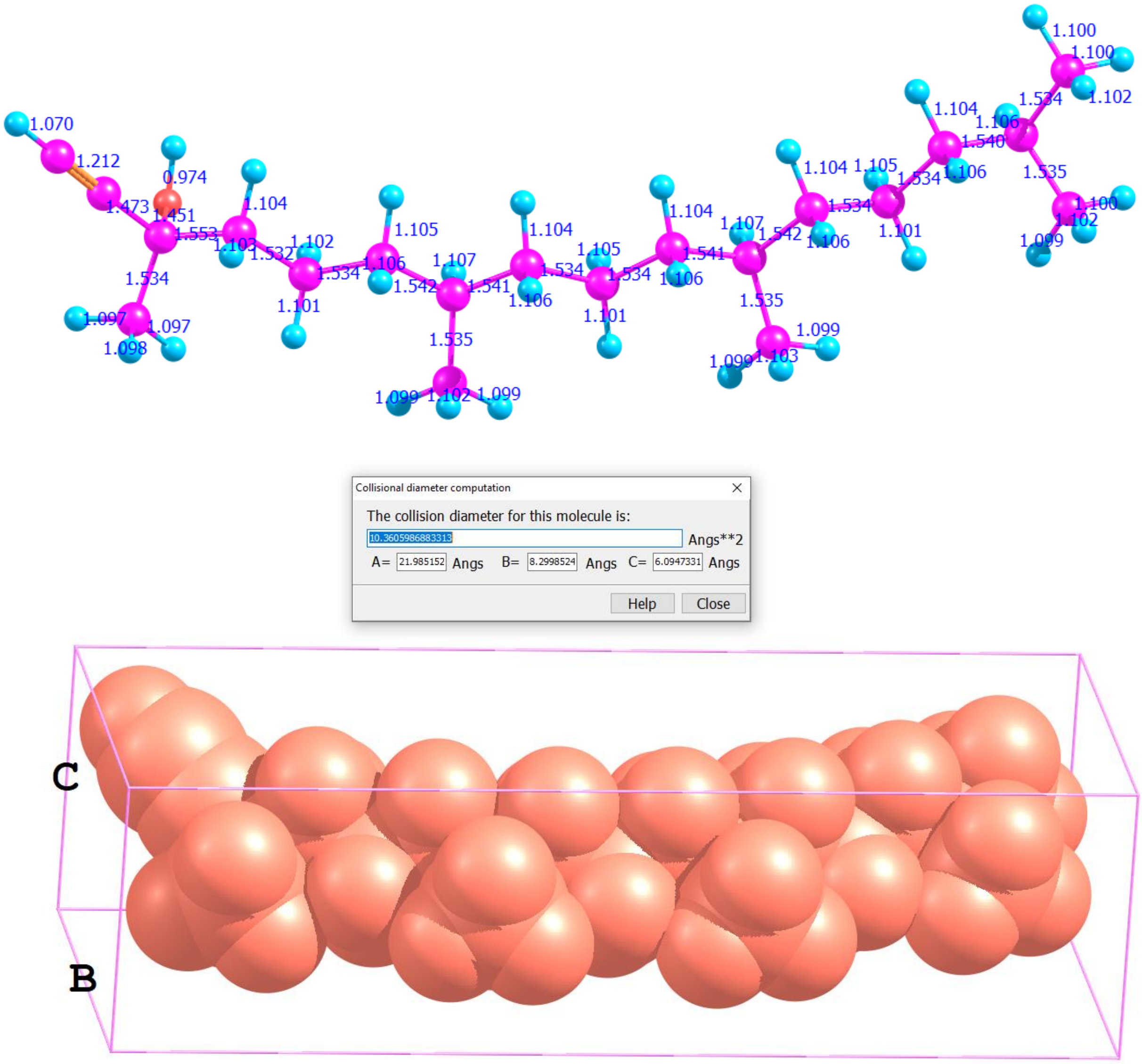

Figure S3.DFT calculation of DHIP.

Table S1. Summary data on physicochemical and catalytic properties of Pd/HPS.

N	Catalyst	HPS SSA, m^2/g	Share of micro-pores, vol.%	D_m of meso-pores, nm	Q_w[1], mL/g	D_m of Pd NPs, nm	Substrate: size (Å) and dipole moment	S_{95}, %	X_{max}, %	R_0, $mol_{sub}/(mol_{Pd}*s)$
1	Pd/C7	420 ± 8	12.1	25	1.18	5.9 ± 2.0, aggregates	MBY 5.9 x 5.9 x 7.1 μ = 1.24 D	96.0	99.9	10.2
2	Pd/PS	650 ± 13	39.3	n.a.	1.23	7.4 ± 2.6, aggregates		96.5	99.0	4.6
3	Pd/OF2	680 ± 14	12.6	25	1.64	1.5 ± 0.5		97.2	99.6	25.4
4	Pd/OF3	710 ± 14	26.6	n.a.	1.57	7.8 ± 3.9, aggregates		95.1	99.9	17.2
5	Pd/OF2	680 ± 14	12.6	25	1.64	1.5 ± 0.5	MNY 5.9 x 6.2 x 14.2 μ = 1.22 D	95.6	98.5	61.8
6	Pd/OF3	710 ± 14	26.6	n.a.	1.57	7.8 ± 3.9, aggregates		94.2	97.1	16.8
7	Pd/OF2	680 ± 14	12.6	25	1.64	1.5 ± 0.5	DHIP 6.1 x 8.3 x 22.0 μ = 1.05 D	92.9[2]	90.3	48.4
8	Pd/OF3	710 ± 14	26.6	n.a.	1.57	7.8 ± 3.9, aggregates		92.5	100	30.4

[1] Q_w – weight-swelling ratio of polymers in toluene;

[2] Selectivity is indicated at 90.3% of MBY conversion;

[3] R_0– initial transformation rate.
